# Antibiotic Resistance and Phylogenetic Diversity of *Escherichia coli* Isolated from Hospital Wastewater in Gabon

**DOI:** 10.3390/microorganisms14050987

**Published:** 2026-04-28

**Authors:** Wilfried Blandin Evoung Chandja, Annicet-Clotaire Dikoumba, Pierre Philippe Mbehang Nguema, Richard Onanga, Gabriel Falque, Yann Mouanga-Ndzime, Sylvain Godreuil, Barthélémy Ngoubangoye

**Affiliations:** 1 Laboratory of Bacteriology, Interdisciplinary Medical Research Centre of Franceville (CIRMF), Franceville P.O. Box 769, Gabon; evoungblandin@gmail.com (W.B.E.C.); dikoumba@hotmail.com (A.-C.D.); yann_mouangandzime@yahoo.fr (Y.M.-N.); genistha@hotmail.com (B.N.); 2 Department of Microbiology, University of Health Sciences and Techniques (USTS), Libreville P.O. Box 19 383, Gabon; 3 Department of Medical Biology, Omar Bongo Ondimba Military Teaching Hospital (HIAOBO), Libreville P.O. Box 20 404, Gabon; 4 Microbiology Laboratory, Research Institute of Tropical Ecology (IRET), Libreville P.O. Box 13354, Gabon; mbehangphilippe@gmail.com; 5 Emerging Viral Diseases Unit, Interdisciplinary Medical Research Centre of Franceville (CIRMF), Franceville P.O. Box 769, Gabon; gabfalque@hotmail.fr; 6 Laboratory of Bacteriology, Montpellier University Hospital Centre, UMR MIVEGEC (IRD, CNRS, University of Montpellier), 34295 Montpellier, France; s-godreuil@chu-montpellir.fr

**Keywords:** hospital wastewater, *Escherichia coli*, phylogenetic groups, ESBL, *bla*CTX-M, One Health, Central Africa and antibiotic resistance

## Abstract

Hospital wastewater represents a critical hotspot for the dissemination of antibiotic resistance genes (ARGs), serving both as an environmental reservoir and a transmission pathway for multidrug-resistant bacteria into receiving ecosystems. The intense antibiotic selective pressure within healthcare facilities promotes the emergence, persistence and amplification of resistant strains, posing substantial risks to public health and environmental integrity. This study aimed to characterize *Escherichia coli* (*E. coli*) isolates recovered from hospital wastewater effluents in multiple cities across Gabon, with emphasis on bacterial loads, antimicrobial resistance patterns and associated genetic determinants. Wastewater samples were aseptically collected from sewer outlets of eleven healthcare facilities distributed across five provinces over a 12-week period, structured into two six-week sampling campaigns to capture temporal variability. A total of 158 bacterial isolates were obtained, among which 49 were confirmed as *E. coli*. Mean concentrations of presumptive *E. coli* ranged from 7.1 × 10^3^ to 1.49 × 10^9^ CFU/mL, indicating substantial microbial contamination of hospital effluents. Antimicrobial susceptibility testing using the Kirby–Bauer disk diffusion method against 19 antibiotics revealed that all isolates exhibited multidrug-resistant phenotypes. Resistance rates were particularly high to β-lactams and third-generation cephalosporins, reaching 90–100% in most facilities, reflecting strong selective pressure and widespread circulation of resistance mechanisms in urban aquatic environments. In contrast, carbapenems and amikacin remained comparatively effective, with resistance levels below 40%, suggesting partial preservation of last-resort therapeutic options. The values of the Multiple Antibiotic Resistance Index (MARI) ranged from 0.21 to 0.84, indicating selection pressure on different classes of antibiotics. Phylogenetic analysis showed a predominance of phylogroup A, traditionally considered commensal but increasingly associated with the spread of resistance. Groups B2, D/E and F proved to be the most resistant. These groups showed marked resistance to first-line antibiotics. The *bla*CTX-M-1 was the most prevalent resistance determinant (66.6%), occurring twice as frequently as *bla*SHV (33.3%), a finding that confirms the significant circulation of extended-spectrum β-lactamase-producing *E. coli*. Overall, these findings highlight hospital wastewater as a major reservoir and dissemination source of multidrug-resistant *E. coli*, underscoring the urgent need for improved wastewater treatment, strengthened antimicrobial stewardship and integrated One Health-based surveillance strategies.

## 1. Introduction

The emergence and spread of antimicrobial resistance (AMR) are now major threats to global public health, compromising the effectiveness of treatments for bacterial infections and significantly increasing morbidity, mortality and associated healthcare costs [1]. In this context, anthropogenic environments, particularly healthcare facilities, hospital effluents, and municipal wastewater treatment plants, are recognized as critical hotspots for the selection, amplification, and spread of antibiotic-resistant bacteria (ARB) and associated resistance genes (ARGs) [2].

Hospital effluents are characterized by a high and diverse load of microbiological and chemical contaminants, resulting in particular from the intensive use of pharmaceutical substances. They contain significant concentrations of drug residues, including radiological contrast agents, disinfectants, and broad-spectrum antibiotics [3]. The co-presence of these compounds exerts strong selection pressure on bacterial communities, promoting the persistence of multi-resistant bacteria and the co-selection of resistance determinants through complex genetic mechanisms [4].

Among these contaminants, antibiotics are a major concern due to their environmental persistence and low removal by conventional wastewater treatment processes. Their accumulation in aquatic environments is closely associated with the emergence and spread of AMR at the interface between human, animal and environmental compartments, in line with the ‘One Health’ conceptual framework [5]. The continued presence of low concentrations of antibiotics in effluents promotes the rapid evolution of bacterial populations, particularly through the horizontal transfer of resistance genes, thereby facilitating the spread of these determinants in urban water networks and natural ecosystems [1].

Hospital wastewater, whether discharged raw or after partial treatment, thus plays a central role in the dispersion of emerging pollutants and resistant bacteria into rivers, soil and groundwater. This issue is particularly concerning in low- and middle-income countries, where water treatment infrastructure is often inadequate or unsuitable for the specific treatment of hospital effluents [6]. In sub-Saharan Africa, hospital effluents are frequently discharged into urban sewerage systems without prior dedicated treatment, increasing the risks of environmental contamination and exposure of human populations [7,8].

Among the bacteria of interest, *E. coli* is one of the species most frequently detected in wastewater due to its ubiquity and its role as an indicator of fecal contamination. Its constant presence in human and animal excreta makes it a relevant tracer of anthropogenic pollution in aquatic environments [9,10,11]. Numerous studies have shown that *E. coli* dominates the bacterial flora of hospital and urban effluents, confirming its use as a sentinel organism for assessing the microbiological quality of water and the risks associated with antibiotic resistance [12,13].

The phylogenetic background of *E. coli* strains is strongly associated with virulence potential, ecological niche adaptation, and the acquisition of antimicrobial resistance determinants. Furthermore, phylogenetic analyses revealed an over-representation of phylogroups B2 and D among resistant *E. coli* isolates, as these groups are frequently associated with high pathogenic potential and an increased ability to acquire resistance genes [14,15]. The identification of these phylogroups in hospital effluents suggests that they are not only reservoirs of commensal strains, but also of virulent lineages that can spread in urban water networks and natural environments [16].

Extended-spectrum beta-lactamase (ESBL)-producing strains, including *E. coli*, are of particular interest because of their association with treatment failure, prolonged hospitalization and increased mortality [17]. Their presence in hospital effluents reinforces the hypothesis that these environments are major environmental reservoirs of clinically relevant bacteria capable of spreading to the community [18,19].

Despite the growing importance of AMR from a One Health perspective, data on antibiotic resistance and the phylogenetic structure of *E. coli* from hospital effluents remain extremely limited in Central Africa, particularly in Gabon. The lack of integrated environmental surveillance is a major obstacle to assessing the health and environmental risks associated with the spread of multidrug-resistant bacteria in this region [11,20].

Thus, the present study aimed to characterize antibiotic resistance profiles, ESBL production and the distribution of *Escherichia coli* phylogroups isolated from hospital wastewater in several cities in Gabon [21]. This integrated approach aims to better understand the role of hospital effluents as reservoirs and vectors of resistant bacteria, and to provide essential data for the development of AMR surveillance and management strategies from a One Health perspective [22].

## 2. Materials and Methods

### 2.1. Study Sites

Hospital wastewater was collected directly at exit points from hospital sewer sumps before reaching the main sewer network, as well as from inspection chambers and septic tanks of various healthcare services. Treated wastewater (TWW) samples were collected at the outlet of treatment plants before their discharge into groundwater or water bodies.

Sampling was carried out during two collection missions, each lasting six weeks, without taking seasonal variations into account. Samples were collected in sterile glass containers (1000 mL) at a depth ranging from 3 to 5 m. One hundred fifty-eight samples of untreated wastewater were collected from various hospitals at different sampling sites. Surface wastewater samples were taken from a watercourse surrounding the hospital, at a distance of 10 m upstream from its discharge point (site 1). Other samples were collected from different discharge points corresponding to various hospital units, including those dedicated to intensive care and surgery (operating room) (site 2), maternity (site 3), emergency services (site 4), pediatrics (site 5), gynecology and obstetrics (site 6), internal medicine (site 7), general medicine (site 8), and orthopedics and surgery (site 9). Samples were transported in isothermal containers at 4 °C and analyzed within two hours after collection.

### 2.2. Hospital Wastewater Management at Each Data Collection Site

Wastewater management varied significantly between facilities. Some institutions exhibit inadequate maintenance of their treatment systems, while others ensure more appropriate upkeep.

#### Culture and Isolation of *E. coli* with Serial Dilution of the Sample

In a cryotube containing 9 mL of sterile water, 1 mL of the sample was added. The mixture was thoroughly agitated. This was the 10^−1^ dilution. From the 10^−1^ dilution, 1 mL was added to 9 mL of sterile water and thoroughly mixed. This was the 10^−2^ dilution. By repeating the same procedure, the dilution was carried out up to 10^−5^. This procedure was performed in a laminar airflow cabinet. Then, 1000 μL of the 10^−1^, 10^−2^, 10^−3^, 10^−4^, and 10^−5^ dilutions was plated in duplicate using the spread plate method on Eosin Methylene Blue (EMB) agar (BioMérieux, Marcy-l’Étoile, France). The inoculated agar plates were incubated for 24 h at 37 °C, after which colonies generally displaying a metallic green sheen were presumed to be *E. coli* isolates. After 24 h, Gram staining and oxidase tests were performed on the presumptive *E. coli* isolates. The presumptive isolates were preserved in a mixture containing 30% glycerol and 70% PBS at −20 °C until further use.

### 2.3. Isolation and Identification of Colonies (E. coli)

Within the Bacteriology Laboratory of the Interdisciplinary Medical Research Centre of Franceville (CIRMF), 100 µL of each cryopreserved sample was carefully inoculated onto selective Eosin Methylene Blue (EMB) agar plates (BioMérieux, Marcy-l’Étoile, France) and incubated at 37 °C for 24 h.

After incubation, distinct colonies, differentiated by their morphology and pigmentation, were collected and re-inoculated onto fresh plates following the same procedure, and then incubated under identical conditions to obtain pure cultures. Purified colonies were subsequently subjected to comprehensive biochemical identification. Colonies exhibiting a metallic green sheen, characteristic of certain Enterobacteriaceae, were identified using API 20E strips (BioMérieux, Marcy-l’Étoile, France) and the automated VITEK^®^ 2 Compact 15 system (BioMérieux, Marcy-l’Étoile, France), enabling accurate and rapid species-level identification.

### 2.4. Evaluation of Antibiotic Resistance in Isolated E. coli

The antibiotic susceptibility of *E. coli* isolates was assessed using the disk diffusion method (Kirby–Bauer) on Mueller–Hinton (MH) agar (BioMérieux, Marcy-l’Étoile, France), in accordance with the guidelines by the Clinical and Laboratory Standards Institute (CLSI) [23]. A panel of 19 antibiotics belonging to eleven distinct classes was employed. Briefly, MH agar plates were inoculated with a standardized bacterial suspension (0.5 McFarland) prepared from 24 h primary cultures of each *E. coli* isolate. Antibiotic-impregnated disks (Oxoid, Basingstoke, Hampshire, UK) were firmly placed onto the surface of the inoculated agar. The plates were then incubated at 37 °C for 24 h. The tested antibiotics included amoxicillin–clavulanic acid (AML, 30 µg), ampicillin (AMP, 10 µg), aztréonam (ATM, 30 µg), amoxicilin (AMC), tetracycline (TE, 30 µg), amikacin (AK, 30 µg), gentamicin (GEN, 10 µg), ceftazidime (CAZ, 30 µg), cefepime (FEP, 30 µg), cefoxitin (FOX, 30 µg), cefotaxime (CTX, 30 µg), imipenem (IMI, 10 µg), ertapenem (ETP, 10 µg), meropenem (MRP, 10 µg), ofloxacine (OFX, 5 µg), nalidixic acid (NA, 30 μg), fosfomycin (FOS, 200 µg), ticarcilin/clavulanic acid (TTC, 85 μg) and nitrofurantoin (F, 300 μg). After 24 h incubation, the zones of inhibition were measured in millimeters using a caliper, and the results were interpreted as ‘Resistant (R), Intermediate (I) or Sensitive (S)’ using the CLSI threshold.

### 2.5. Characterization of Multidrug Resistance: Antibiotic Resistance Phenotype (MARP) and Multiple Antibiotic Resistance Index (MARI)

Isolates exhibiting resistance to at least three of the six different classes of tested antimicrobials were considered to be multidrug-resistant (MDR). The analysis focused on multidrug resistance profiles, the number of antibiotics to which the isolates were resistant, and the diversity of observed phenotypic profiles. The MARI (Multiple Antibiotic Resistance Index) of each multidrug-resistant isolate was calculated using a mathematical equation described by Krumperman [22]. The mathematical expression is given as:

**MARI = x/y**

In this context, “x” represents the number of antibiotics to which a bacterial species showed resistance, while “y” is the total number of antibiotics to which the bacterial species was exposed. A MARI value greater than 0.2 is an indicator of intensive antibiotic use in that region and can be used to predict high-risk areas for antibiotic resistance [24,25].

### 2.6. Isolation of Extended-Spectrum Beta-Lactamase (ESBL)-Producing E. coli

Confirmation of extended-spectrum beta-lactamase (ESBL) production was performed using the double-disk diffusion test on Mueller–Hinton agar, in accordance with the Clinical and Laboratory Standards Institute guidelines [24]. This method involves placing an amoxicillin–clavulanic acid (AMC) disk at the center of the plate and, at a 90 °C angle and approximately 3 cm away, disks of third- or fourth-generation cephalosporins—cefotaxime (CTX), ceftazidime (CAZ), cefepime (FEP)—as well as a monobactam disk, namely aztreonam (ATM). ESBL production is evidenced by the appearance of a characteristic synergy pattern, often described as a “champagne-cork” shape [26,27].

A single colony from each purified strain was then selected and grown in brain heart infusion broth to ensure bacterial proliferation. Each isolate was assigned a unique identification number and preserved at −20 °C in a solution consisting of 30% glycerol and 70% phosphate-buffered saline (PBS) for further analysis.

### 2.7. Molecular Characterization and Resistance Gene Screening in E. coli

*E. coli* DNA was extracted using a standard heat-lysis protocol [28,29] and quantified with a Qubit fluorometer (Invitrogen, Thermo Fisher Scientific, Carlsbad, CA, USA). Gene detection, including phylogroups, was performed by agarose gel electrophoresis (2%, Sigma-Aldrich, St. Louis, MO, USA) stained with ethidium bromide, using a 100 bp DNA ladder (Invitrogen by Thermo Fisher Scientific, Carlsbad, CA, USA) as reference. Electrophoresis was carried out in 1× TAE buffer at 100 V for 1 h, and bands were visualized with a Uvipro-Silver Gel Documentation System (Uvitec, Cambridge, UK). PCR reactions were performed in a final volume of 25 μL containing HotStarTaq 10× PCR Buffer (2.5 μL), dNTPs (0.5 μL, 10 mM), forward primer, reverse primer (Table 1), nuclease-free distilled water (Invitrogen, Thermo Fisher Scientific, Carlsbad, CA, USA), and a 5 μL DNA template. Amplifications were carried out on a SimpliAmp™ Plus thermocycler (Applied Biosystems, Thermo Fisher Scientific, Carlsbad, CA, USA).

Different resistance determinants were assayed for in the targeted bacterial isolates showing full or intermediate resistance. Three resistance genes that encode β-lactamases, various variants of ESBL, and resistance gene determinants were assayed for in multiplex PCR protocols, as reported by [31]. The reactions were performed as singleplex in a total volume of 25 μL, using 5 μL of cell lysate as the DNA template, 10 pmol of each of the forward and reverse primers, 12 μL of the EmeraldAmp MAX PCR Master Mix (TaKaRa, Tokyo, Japan) and 6 μL of PCR-grade water. Each gene, with the primer set used and its expected molecular size, is described in Table 2. Amplifications were carried out on a SimpliAmp™ Plus thermocycler (Applied Biosystems, Thermo Fisher Scientific, Carlsbad, CA, USA). The thermal cycling conditions for the PCR assays were as follows: initial denaturation at 94 °C for 7 min, followed by 30 cycles of 94 °C for 40 s, 60 °C for 40 s, and 72 °C for 1 min, and then a final elongation step at 72 °C for 10 min.

### 2.8. Data Analysis

The software Excel was used for data analysis, including calculations of the prevalence of *E. coli*-carrying genes for detecting phylogenetic groups and pathotypes, the prevalence of antimicrobial resistance and confidence intervals. The Multiple Antimicrobial Resistance Index was calculated following Krumperman’s methods [24].

#### Multivariate and Statistical Analysis

Phenotypic antibiotic resistance profiles were standardized (centered and scaled) prior to multivariate analysis. Principal component analysis (PCA) was conducted using the scikit-learn library in Python (3.12.2.) to explore the underlying structure of multidrug resistance patterns.

To formally assess group-level differences, Permutational Multivariate Analysis of Variance (PERMANOVA) was performed using the sci kit-bio package with 999 permutations. Euclidean distance matrices were used to maintain geometric consistency with the PCA representation. The pseudo-F statistic was computed to quantify the ratio of between-group to within-group multivariate variance.

To ensure the validity of PERMANOVA assumptions, homogeneity of multivariate dispersion (PERMDISP) was evaluated prior to interpretation of group differences. Categories with severe class imbalance and subgroups containing fewer than four isolates (n < 4) were excluded to minimize bias in variance estimation and permutation testing. A significance threshold of *p* < 0.05 was applied.

## 3. Results

### 3.1. Management and Analysis of Hospital Wastewater in Gabon

**Figure 1:**
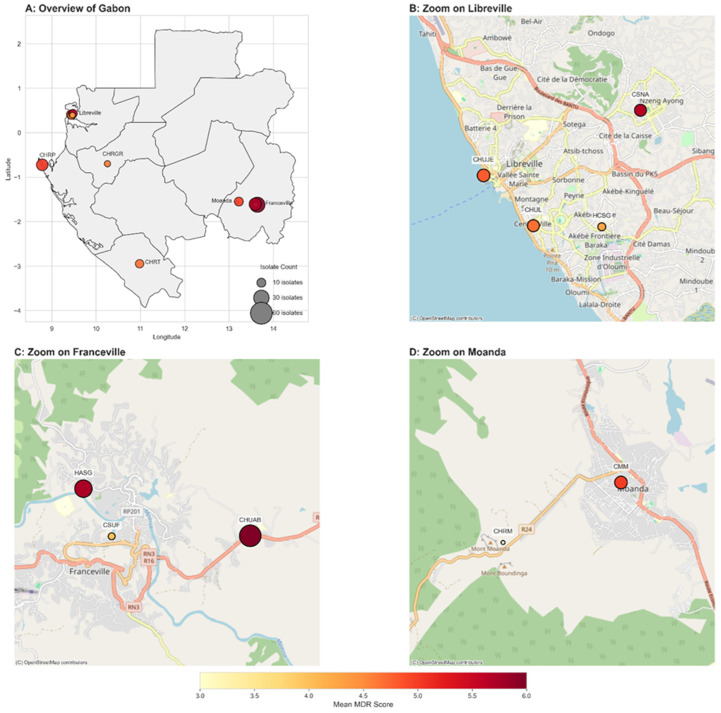
Location of sampling sites (geospatial distribution of MDR scores in Gabonese facilities). Hierarchical geospatial analysis of multidrug resistance intensity across Gabon. The composite figure maps the mean multidrug resistance (MDR) score for isolates from 11 distinct healthcare facilities. In all panels, each point represents a facility, with its color corresponding to the mean MDR score (from yellow for low to red for high intensity) and its size scaled proportionally to the number of isolates sampled. (**A**) The national overview map displays the location of all sampling sites against Gabon’s provincial boundaries, with labels indicating either the city name for urban clusters or the individual facility name for isolated sites. (**B**–**D**) High-resolution insets provide street-level views of the urban areas with the highest sampling density, including Libreville, Franceville, and Moanda, allowing for the precise localization of individual facilities. This multi-scale visualization facilitates the identification of both broad geographic patterns and specific local hotspots of antibiotic resistance, providing critical data for targeted surveillance strategies.

The healthcare facilities (n = 11) from which wastewater samples were collected in this study are distributed across five (5) of the nine (9) provinces of the country (Figure 1). In certain university hospital centers (CHUs), such as Libreville University Hospital (CHUL) (Libreville, Estuaire), Mother and Child University Hospital Centre, Jeanne Ebori Foundation (CHUJE) (Libreville, Estuaire), and Amissa BONGO University Hospital Centre (CHUAB) (Franceville, Haut-Ogooué), a dual filtration system is in place, ultimately discharging wastewater into community water bodies or the groundwater table. However, in other healthcare facilities, namely the Sino-Gabonese Friendship Hospitals (HCSG in Libreville and HASG in Franceville), the Regional Hospital Centers (George RAWIRI Regional Hospital Centre (CHRGR) in Lambaréné, Tchibanga Regional Hospital Centre (CHRT) in Tchibanga, and Port-Gentil Regional Hospital Centre (CHRPG) in Port-Gentil), and some medical centers (Nzeng-Ayong Health Centre (CSNA) in Libreville, Franceville Urban Health Centre (CSUF) in Franceville and Moanda Medical Centre (CMM) in Moanda), wastewater is neither mechanically nor biologically treated due to the absence of technologies such as activated sludge systems or chemical treatment processes. Furthermore, this wastewater is not disinfected through chlorination or UV exposure before being discharged into municipal networks or natural water bodies, despite the recommendations of the Thematic Center for Inland, Coastal, and Marine Waters (ICM) in partnership with the European Environment Agency (EEA).

### 3.2. Isolation and Identification of E. coli

After cultivation on solid media, followed by isolation and biochemical identification, a total of 49 *E. coli* isolates were recovered from 158 wastewater samples collected from the target hospitals.

### 3.3. Enumeration of Isolates

The total number of *E. coli* in wastewater samples discharged from hospital departments ranged from 7.1 × 10^3^ to 1.49 × 10^9^ CFU/mL, whereas samples collected from the surrounding hospital environment showed lower counts, ranging from 1.8 × 10^3^ to 3.0 × 10^5^ CFU/mL. Overall, effluents directly discharged from hospital services exhibited relatively higher bacterial loads compared to runoff waters from healthcare facilities, indicating that wastewater treatment plants were not effective in eliminating bacterial contaminants.

### 3.4. Geographical Distribution of E. coli Isolates

**Figure 2:**
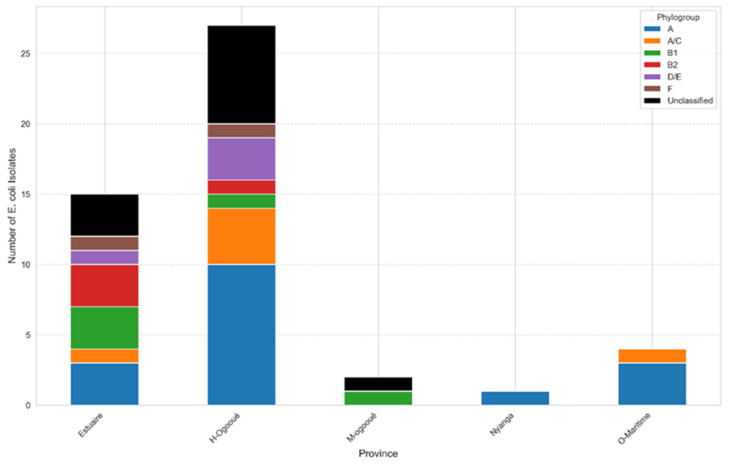
Distribution and phylogroup composition of *E. coli* by province. Several factors may account for these differences. The high concentration observed in Haut-Ogooué and Estuaire could be linked to higher population density, increased hospital activity, or greater intensity of sampling campaigns in these provinces. Conversely, the lower counts recorded in Moyen-Ogooué, Nyanga, and Ogooué-Maritime may reflect reduced bacterial circulation, lower selection pressure, or more limited sampling in these areas.

The distribution of *E. coli* isolates across the provinces of Gabon reveals notable variations between the regions studied, as illustrated in Figure 2. The Haut-Ogooué province recorded the highest number of isolates, with 27 strains, representing the largest proportion of all collected samples. The Estuaire province ranked second, with 15 isolates, also indicating a high prevalence.

In contrast, the Moyen-Ogooué, Nyanga, and Ogooué-Maritime provinces showed considerably lower numbers, with two, one, and four isolates, respectively. These findings highlight a marked disparity in the distribution of strains among the different regions.

### 3.5. Prevalence of Escherichia coli Phylogroups

Among the 49 *E. coli* isolates analyzed, phylogroup A was predominant, accounting for 34.7% of the strains (n = 17/49). It was followed, in decreasing order of frequency, by phylogroups A/C (12.2%; n = 6/49), B1 (10.2%; n = 5/49), D/E (8.16%; n = 4/48), B2 (4.08%; n = 2/49), B2″ (4.08%; n = 2/49), and finally F (4.08%; n = 2/49), and 11 *E. coli* isolates (22.45%; 11/49) were indeterminate to phylogroups. The detailed distribution of these proportions is presented in Table 3.

### 3.6. Distribution of Phylogroup E. coli Isolates

**Figure 3:**
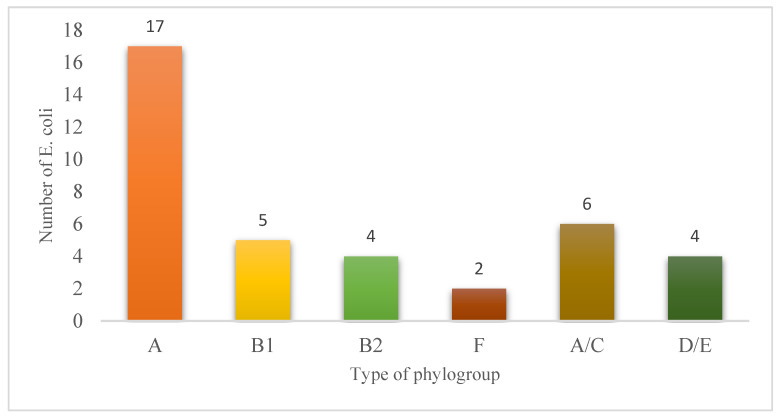
Classification of *E. coli* according to phylogroup. This distribution suggests that certain phylogenetic lineages, particularly group A, may be better adapted to local ecological conditions or associated with more frequent sources of infection. The lower numbers observed in the other groups may reflect either reduced circulation in the studied environment or a more specific geographical or ecological distribution.

Among the *E. coli* phylogroups identified (Figure 3), group A was predominant with 17 isolates, followed by A/C (6 isolates) and B1 (5 isolates). Groups B2 and D/E each accounted for four isolates, while F was the least represented, with only two isolates.

### 3.7. Distribution of Multidrug Resistance Scores by Escherichia coli Phylogroups

**Figure 4:**
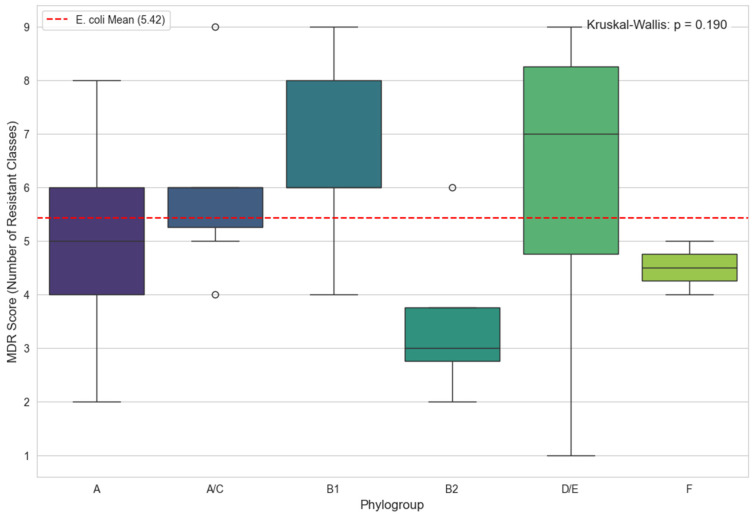
Multidrug resistance (MDR) score distribution among *E. coli* phylogroups. The circles visible on this boxplot represent outliers. These are individual observations whose resistance scores (MDR) fall outside the interval defined by 1.5× the interquartile range (IQR). These points therefore indicate isolates with an exceptionally higher or lower resistance profile compared to the main distribution of the phylogroup concerned.

A comparative analysis of multidrug resistance (MDR) scores was performed for the different Escherichia coli strains classified according to their phylogroups (Figure 4). The MDR score, representing the number of antibiotic classes to which each strain exhibited resistance, ranged overall from 1 to 9, with a global mean of 5.42 (red dashed line). Phylogroup A displayed a median score of approximately 5, similar to the overall mean, but with a wide range from two to eight resistant classes. Phylogroup A/C showed lower variability, with most strains grouped around 5–6 resistant classes. Phylogroup B1 exhibited one of the highest median scores (around 7), with a range from four to nine classes, indicating a marked tendency toward multidrug resistance.

In contrast, phylogroup B2 presented the lowest median score (≈3), with a narrow range of two to four resistant classes, reflecting a resistance profile well below the global mean. Similar to B1, phylogroup D/E showed a high median score (≈7) but with considerable variability, with some strains exhibiting low resistance (score = 1) and others being highly multidrug-resistant (score = 9). Finally, phylogroup F had a moderate median score (≈4.5) and low variability (four to five classes). A non-parametric Kruskal–Wallis test revealed no statistically significant differences in MDR scores between phylogroups (*p* = 0.190).

### 3.8. Antimicrobial Susceptibility

In this study, the resistance profile revealed extremely high rates of resistance to classical beta-lactams (ampicillin, amoxicillin, and ticarcillin–clavulanic acid) and third-generation cephalosporins (cefotaxime, ceftriaxone, and ceftazidime), as well as to aztreonam and tetracycline, with resistance approaching 90–100% in most healthcare facilities. In contrast, carbapenems (imipenem, meropenem, and ertapenem) and amikacin retained relatively preserved activity, with resistance rates generally below 40%. Gentamicin showed intermediate and site-dependent patterns, while cefepime, fosfomycin, and nitrofurantoin exhibited moderate resistance (40–60%). Comparative analysis revealed a higher prevalence of resistance in university hospitals (CHUL, CHUJE, and CHUAB) compared to peripheral facilities (CMM, CSUF, and CSNA).

### 3.9. Distribution of Antibiotic Resistance in Healthcare and Community Wastewater

**Figure 5:**
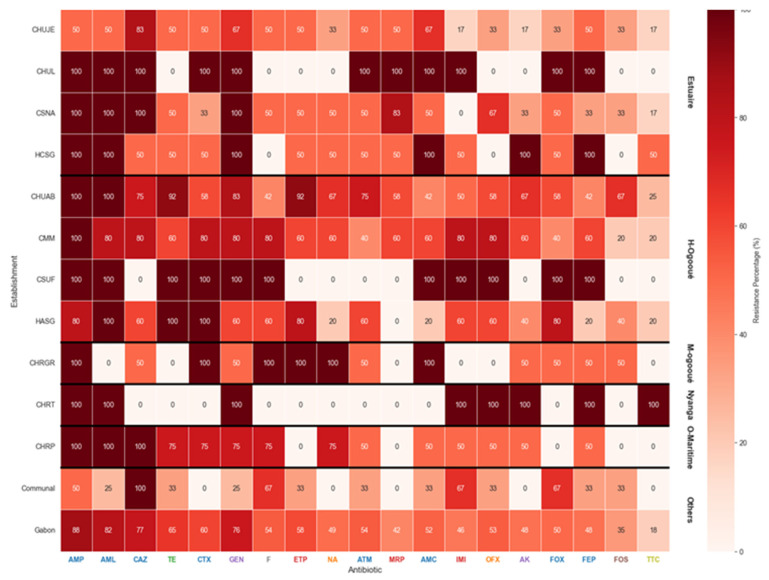
Antibiotic resistance profile by establishment (grouped by province) for *E. coli*. Legend: AMP: Ampicillin; AML: Amoxicillin–Clavulanic Acid; CAZ: Ceftazidim; TE: Tetracycline; CTX: Cefotaxim; GEN: Gentamicin; F: Nitrofurantoin; ETP: Ertapenem; NA: Nalidixic Acid; ATM: Artreonam; MRP: Meropenem; AMC: Amoxicillin; IMI: Imipenem; OFX: Ofloxacin; AK: Amikacin; FOX: Cefoxitin; FEP: Cefepime; FOS: Fosfomycin; TTC: Ticarcillin–Clavulanic Acid. The color indicates the average antibiotic resistance (MDR) score, from low (white, 0%) to high (dark red, 100%).

The distribution of *E. coli* resistance rates across different antibiotics and healthcare facilities is presented in Figure 5. A consistently high level of resistance was observed to β-lactam antibiotics, particularly ampicillin and amoxicillin, with resistance rates approaching 100% in nearly all institutions. Similarly, a substantial proportion of isolates exhibited resistance to combinations with β-lactamase inhibitors, including amoxicillin–clavulanate and ticarcillin–clavulanate, indicating the widespread presence of β-lactamase-mediated resistance.

Resistance to third-generation cephalosporins (cefotaxime, ceftazidime, and ceftriaxone) and cephamycins (cefoxitin) was also frequent, though variability was noted between hospitals. These findings suggest a strong circulation of extended-spectrum β-lactamase (ESBL)-producing *E. coli* across different clinical settings. In contrast, resistance to carbapenems (imipenem, ertapenem, and meropenem) remained relatively low, although sporadic resistant isolates were detected, highlighting the potential emergence of carbapenemase producers.

Among aminoglycosides, resistance to gentamicin and amikacin showed heterogeneous patterns, with some facilities reporting elevated resistance while others displayed lower levels. This variability may reflect differences in aminoglycoside usage policies across healthcare institutions. Resistance to tetracycline, nitrofurantoin, and fosfomycin was moderate to high, with fosfomycin showing comparatively lower resistance levels in most facilities, suggesting its potential usefulness as an alternative therapeutic option.

The data reveal a concerningly high burden of resistance to first-line antibiotics, with widespread dissemination of ESBL phenotypes, while carbapenems remain largely preserved. The inter-hospital variability observed underscores the influence of local antibiotic use practices and the need for targeted stewardship interventions.

### 3.10. Distribution of Antibiotic Resistance Patterns in E. coli Phylogroups Isolated from Hospital Wastewater

**Figure 6:**
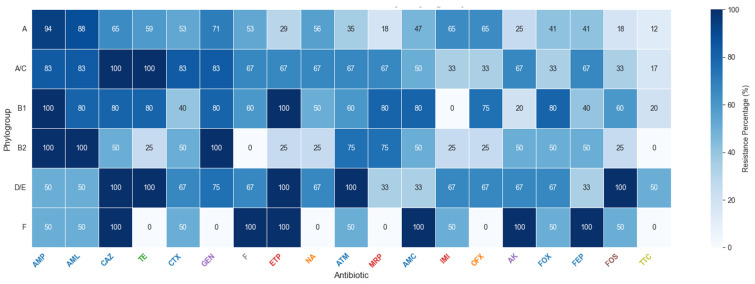
Antibiotic resistance profile of *E. coli* strains stratified by phylogroup. The heatmap displays the percentage of resistant isolates for each antibiotic (columns) within each phylogroup (rows). The color indicates the average antibiotic resistance (MDR) score, from low (white, 0%) to high (dark blue, 100%).

Analysis of the heatmap (Figure 6) showed high overall resistance of *E. coli* isolates, with beta-lactams (ampicillin, amoxicillin, ceftazidime, and cefotaxime) exceeding 60% resistance across all phylogroups. B1 and B2 displayed the broadest multidrug resistance, including near-complete resistance to ampicillin, amoxicillin, ertapenem, and fluoroquinolones, suggesting ESBL and carbapenemase genes. D/E also exhibited strong resistance to cephalosporins and amoxicillin–clavulanic acid, while A and A/C remained largely resistant to beta-lactams (≈50–80%). F showed a mixed pattern, with 100% resistance to ceftazidime, fosfomycin, and cefoxitin but lower resistance to gentamicin, imipenem, and ofloxacin.

The data reveal a high prevalence of multidrug-resistant strains, including carbapenem resistance, highlighting intense selective pressure in hospital wastewater and the need for strengthened surveillance and antibiotic stewardship measures.

### 3.11. Analysis and Interpretation of PCA Results

This figure investigates the underlying structure of antibiotic resistance in *E. coli* isolates through a multi-panel analysis. (A) The scree plot indicates that the first two principal components capture 30.72% of the total variance in the dataset. (B, C, D) These biplots display the distribution of isolates (points) along the first two principal components, with vectors representing the influence of each antibiotic. To assess group separation, 95% confidence ellipses are drawn for each stratum. The same dataset is plotted in each panel, colored by (B) province, (C) collection environment (‘Milieu’), and (D) facility (‘Nom de Hopital’). The extensive overlap of the confidence ellipses across all three methods of stratification visually confirms the absence of distinct, statistically separable clusters.

**Figure 7:**
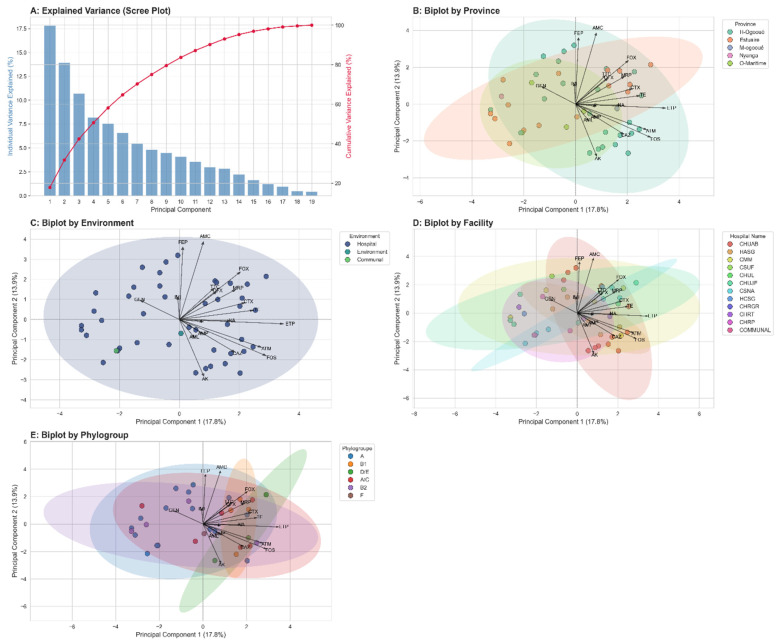
Stratified principal component analysis of *E. coli* resistomes. (**A**) The variance explained by each principal component. The first two components (PC1 and PC2) account for 17.8% and 12.9% of the total variability, respectively, representing approximately 30.7% combined. While the biplots in each panel visually display extensive overlapping of confidence ellipses, suggesting high intra-group variance, a more quantitative analysis reveals significant underlying structures. The dataset is plotted in each panel, colored by (**B**) province, (**C**) collection environment, (**D**) facility, and (**E**) phylogroup. The extensive overlap of the confidence ellipses across all three methods of stratification visually confirms the absence of distinct, statistically separable clusters.

Figure 7A presents the variance explained by each principal component. The first two components (PC1 and PC2) account for 17.8% and 12.9% of the total variability, respectively, representing approximately 30.7% combined. Although this proportion is not very high, it is sufficient to distinguish general trends and visualize clusters of isolates based on their resistance profiles.

Figure 7B illustrates the distribution of isolates according to provinces. Distinct clusters are observed, indicating that certain resistance profiles are more prevalent in specific regions, such as Haut-Ogooué and Estuaire.

Figure 7C compares isolates based on their environmental origin: hospital, community, or environment. Hospital isolates occupy a broader area of the plot, reflecting greater diversity in resistance profiles. In contrast, isolates from the community and environment are more tightly clustered, indicating more homogeneous profiles.

Figure 7D highlights the distribution of isolates according to healthcare facilities. Some hospitals, such as CHUL and CHRGR, exhibit distinct profiles compared to others.

Finally, Figure 7E shows the distribution of isolates according to their phylogroups. Certain groups, such as B2 and D/E, are associated with more pronounced resistance profiles, whereas others, such as A or B1, appear more dispersed. This trend suggests that specific *E. coli* phylogroups play a central role in the dissemination of resistance genes, reinforcing their epidemiological significance.

The PCA highlights notable differences in the resistance profiles of *E. coli* isolates according to province, origin, healthcare facility, and phylogroup. These findings confirm that resistance is not uniformly distributed but is shaped by multiple factors related to both the environment and the genetic characteristics of the strains.

Although PCA biplots revealed substantial visual overlap between confidence ellipses, PERMANOVA identified statistically significant centroid separation for several grouping variables.

Geographical stratification showed significant differences in resistance profiles between adequately represented provinces (pseudo-F = 1.59, *p* = 0.023) (Figure 7B). Similarly, when restricting analysis to healthcare facilities with N ≥ 4 isolates, facility-level clustering was detected (pseudo-F = 1.32, *p* = 0.035) (Figure 7D), indicating localized selective pressures influencing phenotypic resistance signatures. A significant association was also observed between phylogenetic background and multidrug resistance structure (pseudo-F = 1.48, *p* = 0.029) (Figure 7C), supporting the contribution of genetic lineage to resistance pattern variability.

Despite statistical significance, the relatively low pseudo-F values reflect high intra-group heterogeneity, consistent with the broad dispersion observed in PCA space. PERMDISP analysis did not indicate significant differences in dispersion between groups, suggesting that PERMANOVA results primarily reflect centroid separation rather than variance heterogeneity. Thus, although factors such as phylogeny, geography, and the specific healthcare facility significantly shape resistance profiles, multidrug-resistant *E. coli* strains maintain a heterogeneous and universally widespread circulation.

### 3.12. MARI of Targeted Members of the Enterobacteriaceae (E. coli) Group

The MARP and MARI profiles of *Escherichia coli* are summarized in Table 4. All isolates exhibited resistance to at least three of the nineteen antibiotics tested. The highest resistance level was recorded in strains from Haut-Ogooué, which were resistant to 16 antibiotics. MARI values ranged from 0.21 to 0.84, exceeding the critical threshold of 0.2. Most resistance phenotypes were unique.

### 3.13. ESBL Resistance Genes

ESBL-encoding genes were detected in all 21 ESBL-producing *E. coli* isolates. The *bla*CTX-M-1 gene was the most prevalent antimicrobial resistance gene (ARG), identified in 14 out of 21 isolates (66.6%), followed by *bla*SHV (7/21; 33.3%), while *bla*TEM was not detected in any of the isolates analyzed (0/21; 0%). Co-occurrence of *bla*CTX-M with other ESBL genes (*bla*TEM or *bla*SHV) was observed in one multidrug-resistant (MDR) isolate (1/21; 4.8%) in this study.

## 4. Discussion

The *E. coli* strains isolated from hospital effluents exhibited high resistance levels, highlighting an alarming trend in antimicrobial selective pressure in the study area. The fact that all isolates exhibited resistance to at least three antibiotics belonging to three different families indicates the widespread presence of multidrug-resistant strains [34]. Isolates from Haut-Ogooué displayed the highest resistance profile, with resistance to 16 out of 19 antibiotics tested, suggesting either intense antibiotic use or dissemination of highly resistant clones [35,36] in this province.

### 4.1. High Prevalence of Multidrug-Resistant E. coli in Hospital Wastewater

The elevated Multiple Antibiotic Resistance Index (MARI) values, ranging from 0.21 to 0.84, exceed the critical threshold of 0.2, which is widely used as an indicator of environments with high antibiotic exposure. These values reflect a strong selective pressure associated with the frequent use of antibiotics in healthcare centers. Similar MAR indices have been reported in hospital wastewater studies conducted in India [37], Poland [38], Ethiopia [39], and other countries, which often report MAR values ≥ 0.5 for *E. coli* from hospital effluents, reinforcing the notion that healthcare effluents globally represent hotspots for the emergence and persistence of multidrug resistance.

A MARI greater than 0.2 is generally considered an indicator of high-risk environments where antibiotics are excessively or inappropriately used; these results suggest significant selective pressure in the studied regions [24]. The observation that most resistance phenotypes were unique suggests the involvement of multiple independent resistance mechanisms rather than the clonal expansion of a single lineage [36].

Taken together, these findings underscore the urgent need for continuous surveillance of antimicrobial resistance in Gabon and for strengthened antimicrobial stewardship policies to limit the emergence and spread of multidrug-resistant *E. coli* [40].

### 4.2. Antibiotic Resistance Patterns and Preservation of Last-Resort Agents

The resistance profiles observed reveal extremely high resistance rates to commonly used β-lactams, including ampicillin, amoxicillin, and third-generation cephalosporins, as well as to aztreonam and tetracycline. In most healthcare facilities, resistance levels approaching 90–100% suggest a near-complete loss of efficacy of these antibiotics in the studied settings [41]. These findings are consistent with reports from other sub-Saharan countries, such as Burkina Faso, where ESBL-producing Enterobacterales were predominant in hospital wastewater [42].

In contrast, carbapenems and amikacin retained relatively preserved activity, with resistance rates generally below 40%, corroborating findings from Ghana where these drugs remained effective against multidrug-resistant isolates [38]. Although this observation is reassuring, the detection of carbapenem-resistant isolates remains concerning, as it may signal the early emergence of carbapenemase-producing strains. Given that carbapenems are often considered last-resort antibiotics for severe infections, their compromised effectiveness in environmental reservoirs poses a serious threat to future treatment options. Intermediate resistance (40–60%) was noted for cefepime, fosfomycin, and nitrofurantoin, reflecting variable efficacy across facilities. Similar patterns have been described in West Africa, where resistance prevalence in hospital effluents reached 39% for *E. coli* and 26% for *K. pneumoniae* [43].

Resistance levels varied by facility, with university hospitals (CHUL, CHUJE, and CHUAB) showing the highest rates, likely due to higher antibiotic pressure, while peripheral centers exhibited slightly lower rates. Comparable gradients between tertiary and primary hospitals have been documented in South Africa and Nigeria [44,45].

These findings underscore the need for improved wastewater management to curb the environmental spread of resistant bacteria [46] and for strengthened antibiotic stewardship, particularly in tertiary hospitals, to preserve the efficacy of last-resort agents. Establishing integrated surveillance programs that combine clinical and environmental monitoring is essential to anticipate and contain the dissemination of resistance [47].

### 4.3. Study Context and Importance of E. coli Phylogroups

*E. coli* is used as an indicator of fecal contamination and as a sentinel for the dissemination of antimicrobial resistance determinants (ARDs) in wastewater [48]. Its phylogroups display contrasting profiles: A and B1, which are mostly commensal, as opposed to groups B2 and D, which are more virulent and often involved in extraintestinal infections [49].

In our samples, phylogroups A and A/C were the most frequent, followed by groups B1, B2, D/E, and F. This distribution, dominated by commensal groups (A and B1), differs from the expected pathogenic profile (B2 and D) [50], but is consistent with observations reported in Portugal, Malaysia, and Iraq [51,52]. This suggests that the source of isolates, particularly hospital effluents and surrounding waters, influences the phylogenetic composition of *E. coli* populations and shapes the circulation of resistance genes [53].

### 4.4. Antibiotic Resistance Profiles of E. coli Phylogroups

The heatmap revealed distinct resistance patterns across *E. coli* phylogroups, with B2, D/E, and F emerging as the most resistant. These groups showed marked resistance to first-line antibiotics, including cephalosporins (CAZ and CTX) and carbapenems (MRP and ETP), consistent with previous studies linking them to severe extraintestinal infections and to the carriage of ESBLs and fluoroquinolone resistance genes [54]. In contrast, phylogroup A exhibited the lowest overall resistance, in line with its status as a commensal group with fewer resistance determinants [55]. Phylogroup B1, often considered commensal, showed unexpectedly high resistance to some antibiotics (AMP, TE, and CTX), supporting evidence that this group can acquire resistance and contribute to infections. The combined D/E group also confirmed its pathogenic potential with strong resistance, particularly in urinary tract infections [56]. While these results largely align with global patterns, variations exist. Some studies report regional differences, with phylogroup B2 occasionally less resistant than A or D [57]. Furthermore, resistance is not solely determined by phylogroup but also by mobile genetic elements, particularly plasmids, which facilitate horizontal gene transfer [58]. This may explain why even typically susceptible groups, such as A or B1, can display elevated resistance to specific antibiotics [59].

### 4.5. Phylogenetic Structure and Association with Multidrug Resistance

#### ESBL Gene Distribution and Dominance of *bla*CTX-M

Molecular analysis confirmed the presence of ESBL genes in all ESBL-producing isolates, with *bla*CTX-M-1 being the most prevalent determinant, followed by *bla*SHV. The absence of *bla*TEM is consistent with recent trends indicating a global shift toward CTX-M-type ESBLs, particularly in *E. coli*. The dominance of *bla*CTX-M genes has been widely reported in both clinical [60,61,62] and environmental isolates across Africa, Europe, and Asia, highlighting their successful dissemination and strong selective advantage. In support of our findings, the dominance of *bla*CTM-M in *E. coli* has been reported in aquatic environments [63].

The presence of these genes in hospital wastewater raises concerns about their potential spread to the community through environmental exposure, contaminated water sources, and food chains [64]. Hospital effluents may therefore act not only as reservoirs but also as amplification points for ESBL-producing bacteria, facilitating the transfer of resistance genes to other bacterial species [65].

### 4.6. Public Health Implications and One Health Perspective

From a One Health perspective, the circulation of ESBL-producing *E. coli* in environmental compartments poses risks to human, animal, and ecosystem health [66]. Environmental exposure may contribute to the silent colonization of human populations, complicating infection control efforts and undermining antibiotic stewardship programs [67]. These results emphasize the need for integrated surveillance systems that combine clinical, environmental, and molecular data to better track resistance dynamics and inform targeted interventions [68].

The emergence and spread of antibiotic resistance genes (ARGs) in aquatic environments constitute a major public health challenge, exacerbated by hospital discharges. Recent metagenomic studies highlight that, although treatment systems influence bacterial composition, they do not always succeed in eliminating the abundance of ARGs [69]. Indeed, a comparative analysis between raw and treated effluents shows changes in microbial diversity without neutralizing the resistance signature [70]. This persistence reinforces the need for increased environmental monitoring, particularly through wastewater-based epidemiology (WBE), to better assess the burden of hospital and community resistomes on the overall ecosystem [71].

## 5. Conclusions

This study indicates a substantial presence of multidrug-resistant *E. coli* in hospital and urban effluents in Gabon, with many isolates showing resistance to multiple antibiotics and MARI values exceeding the commonly used critical threshold, suggesting considerable antibiotic selective pressure. Phylogroups B2, D/E, and F exhibited comparatively higher resistance levels, supporting their potential involvement in the dissemination of resistance determinants. By combining phenotypic resistance patterns, phylogenetic characterization, and molecular detection of ESBL-associated genes, the study provides an integrated overview of resistant *E. coli* circulating at the interface between healthcare settings and the surrounding environment. Overall, these findings underscore the importance of continued surveillance of antimicrobial resistance, alongside efforts to enhance antibiotic stewardship and wastewater management, in order to help mitigate the spread of multidrug-resistant strains and sustain treatment effectiveness.

## Figures and Tables

**Table 1:** Primer pairs employed for the detection of genes indicative of specific phylogroups.

PCR Reaction	Target	Primer ID	Primer Sequences (5′-3′)	PCR Product (bp)	Reference
Quadruplex	chuA	chuA.1b	ATGGTACCGGACGAACCAAC	288	[30]
		chuA.2	TGCCGCCAGTACCAAAGACA		
	yjaA	yjaA.1b	CAAACGTGAAGTGTCAGGAG	211	[30]
		yjaA.2b	AATGCGTTCCTCAACCTGTG		
	TspE4.C2	TspE4C2.1b	CACTATTCGTAAGGTCATCC	152	[30]
		TspE4C2.2b	AGTTTATCGCTGCGGGTCGC		
	arpA	AceK.f	AACGCTATTCGCCAGCTTGC	400	[30]
		ArpA1.r	TCTCCCCATACCGTACGCTA		

**Table 2:** List of primer sequences used in multiplex PCR for the determination of ESBL determinants.

Primer ID	Primer Sequences (5′-3′)	PCR Product (bp)
CTX-M-1	GCCCGAGGTGAAGTGGTATC	304
	GTGAAAGCGAACCGARTCTG	
TEM	CGGGAAGCTAGAGTAAGTAGTTCS	458
	AACAGCGGTAAGATCCTTGAGAG	
SHV	CGCTGTTATCGCTCATGGTAA	272
	CGTAGGCATGATAGAAATGGATCTG	

**Table 3:** Distribution of *E. coli* isolates into phylogenetic groups based on the Clermont method [32,33].

Quadruplex Genotype					Number	Frequency (%)
Phylogroup	arpA	chuA	yjaA	TspE4.C2		
A	+	−	−	−	17	34.7
B1	+	−	−	+	5	10.2
B2	−	+	+	−	2	4.08
B2”	−	+	+	+	2	4.08
F	−	+	−	−	2	4.08
A/C	+	−	+	−	6	12.2
D/E	+	+	−	−	4	8.16

Legend: The phylogenetic types **A** (ArpA+, ChuA−, YjaA−, TSPE4.C2−), **B1** (ArpA+, ChuA−, YjaA−, TSPE4.C2+), **B2** (ArpA−, ChuA+, YjaA+, TSPE4.C2−), **B2″** (ArpA−, ChuA+, YjaA+, TSPE4.C2+), **F** (ArpA−, ChuA+, YjaA−, TSPE4.C2−), **A/C** (ArpA+, ChuA−, YjaA+, TSPE4.C2−) and **D/E** (ArpA+, ChuA+, YjaA−, TSPE4.C2−) were observed in this study.

**Table 4:** MAR index profiles in *Escherichia coli* isolated from hospital wastewater.

Resistance Phenotype Profile of *Escherichia coli*	RAM	MARP	MAR Index
ESTUAIRE (n = 15)			0.51
CAZ, NA, F	3	1	0.16
AMP, AML, MRP, GEN	4	1	0.21
CAZ, AMC, FEP, GEN, TE	5	1	0.26
AMP, AML, CAZ, NA, GEN, AK	6	1	0.31
AMP, AML, OFX, MRP, GEN, AK, F	7	1	0.36
AMP, AML, ATM, CAZ, CTX, MRP, GEN	7	1	0.36
AMP, AML, ATM, AMC, FEP, GEN, AK	7	1	0.36
AMP, AML, ATM, CAZ, AMC, FEP, CTX, ETP, AK, F,	10	1	0.53
AMP, AML, ATM, CAZ, AMC, FEP, CTX, FOX, IMI, MRP, GEN	11	1	0.58
AMP, AML, CAZ, AMC, FOX, TTC, OFX, ETP, MRP, GEN, FOS, F, TE	13	1	0.68
AMP, AML, ATM, CAZ, AMC, CTX, FOX, OFX, IMI, ETP, MRP, GEN, FOS, TE	14	1	0.74
ATM, CAZ, AMC, FEP, CTX, FOX, TTC, OFX, NA, ETP, MRP, FOS, F, TE	14	1	0.74
AMP, AML, ATM, CAZ, AMC, FEP, CTX, FOX, OFX, NA, ETP, MRP, GEN, TE	14	1	0.74
AMP AML, CAZ, AMC, FEP, CTX, FOX, TTC, NA, IMI, ETP, MRP, GEN, AK, TE	15	1	0.79
AMP, AML, ATM, AK, AMC, FEP, FOX, OFX, NA, ETP, MRP, GEN, FOS, F, TE	15	1	0.79
**HAUT-OGOOUE (n = 27)**			**0.61**
AMP, AML, OFX, IMI, GEN, F	6	3	0.31
AML, CTX, FOX, ETP, GEN, F, TE	7	1	0.36
AMP, ATM, CAZ, FOX, OFX, IMI, AK, F	9	1	0.47
AMP, AML, CTX, FOX, NA, IMI, ETP, FOS, TE	9	1	0.47
CAZ, AMC, FEP, FOX, IMI, ETP, MRP, FOS, F	10	1	0.53
AMP, AML, ATM, CAZ, FOX, ETP, MRP, AK, FOS, TE	10	1	0.53
AMP, AML, ATM, CAZ, CTX, FOX, OFX, ETP, AK, TE	10	1	0.53
AMP, CAZ, AMC, FEP, CTX, FOX, OFX, IMI, GEN, AK	10	1	0.53
AMP, AML, ATM, CAZ, NA, IMI, ETP, GEN, AK, FOS, TE	11	1	0.58
AMP, AML, AMC, FEP, CTX, FOX, TTC, NA, MRP, GEN, TE	11	1	0.58
AMP, AML, AMC, FEP, FOX, OFX, NA, IMI, ETP, GEN, TE	11	1	0.58
AMP, AML, AMC, FEP, CTX, FOX, OFX, IMI, GEN, F, TE	11	1	0.58
AMP, AML, ATM, CAZ, CTX, OFX, NA, ETP, AK, FOS, F, TE	12	1	0.63
AMP, AML, ATM, CAZ, CTX, FOX, ETP, MRP, GEN, AK, FOS, TE	12	1	0.63
AMP, AML, ATM, CAZ, CTX, IMI, ETP, GEN, AK, FOS, F, TE	12	1	0.63
AMP, AML, ATM, CAZ, AMC, FEP, CTX, OFX, IMI, GEN, F, TE	12	1	0.63
AMP, AML, AMC, FEP, FOX, TTC, OFX, IMI, ETP, MRP, GEN, F, TE	13	1	0.68
AMP, AML, ATM, CAZ, OFX, NA, IMI, ETP, GEN, AK, FOS, F, TE	13	1	0.68
AMP, AML, ATM, CAZ, CTX, FOX, OFX, NA, ETP, MRP, GEN, AK, FOS, TE	14	1	0.74
AMP, AML, CAZ, AMC, FEP, CTX, OFX, NA, ETP, MRP, GEN, AK, F, TE	14	1	0.74
AMP, ATM, CAZ, AMC, FEP, CTX, FOX, OFX, NA, IMI, ETP, MRP, F, TE	14	1	0.74
AMP, AML, ATM, CAZ, AMC, FEP, CTX, FOX, OFX, NA, IMI, ETP, MRP, GEN, F	15	1	0.79
AMP, AML, ATM, CAZ, CTX, FOX, TTC, OFX, IMI, ETP, GEN, AK, FOS, F, TE	15	1	0.79
AMP, AML, ATM, CAZ, CTX, TTC, NA, IMI, ETP, MRP, GEN, AK, FOS, F, TE	15	1	0.79
AMP, AML, ATM, CAZ, AMC, FEP, CTX, TTC, OFX, NA, ETP, MRP, GEN, AK, FOS, TE	16	1	0.84
**MOYEN-OGOOUE (n = 2)**			**0.47**
AMP, AMC, CTX, ETP, GEN, F	6	1	0.31
AMP, ATM, CAZ, AMC, FEP, CTX, FOX, NA, ETP, AK, FOS, F	12	1	0.63
**NYANGA (n = 1)**			**0.42**
AMP, AML, FEP, TTC, OFX, IMI, GEN, AK	8	1	0.42
**OGOOUE-MARITIME (n = 4)**			**0.46**
AMP, AML, NA, IMI, GEN, AK, F	7	1	0.37
AMP, AML, CAZ, AMC, FEP, CTX, IMI, GEN, TE	9	1	0.47
AMP, AML, ATM, CAZ, NA, GEN, AK, F, TE	9	1	0.47
AMP, AML, ATM, CAZ, AMC, CTX, OFX, NA, F, TE	10	1	0.53
**The MAR index at the national level for the study**			**0.49**

## Data Availability

The raw data supporting the conclusions of this article will be made available by the authors on request.
